# Multiple bladder calculi after radical prostatectomy due to bladder neck stenosis in a patient with hypertrophic scar: A case report

**DOI:** 10.1016/j.ijscr.2023.108829

**Published:** 2023-09-14

**Authors:** Mathew Yamoah Kyei, Maxwell Agyapong Nyinah, Ali Ayamba, Josephine Mpomaa Kyei, James Edward Mensah

**Affiliations:** a Department of Surgery and Urology, University of Ghana Medical School, Box GPO 4236, Accra, Ghana; b Korle bu Teaching Hospital, Box KB77, Korle bu, Accra, Ghana; c University of Health and Allied Sciences, PMB 31, Ho, Ghana; d School of Nursing and Midwifery, University of Ghana, Box LG 43, Legon, Accra, Ghana; e Department of Surgery and Urology, University of Ghana Medical School, Box GPO 4236, Accra, Ghana

**Keywords:** Prostate cancer, Radical prostatectomy, Bladder calculi, Bladder neck stenosis, Hypertrophic scar, Case report

## Abstract

**Introduction and importance:**

Bladder calculi after radical prostatectomy is rare and usually associated with migrated clips into the bladder forming a nidus. We present a patient with multiple bladder calculi resulting from bladder neck stenosis after radical prostatectomy causing bothersome lower urinary tract symptoms. He had an associated hypertrophic scar.

**Case presentation:**

A 60-year-old man of African ancestry presented with recent onset of irritative urinary symptoms three years after radical prostatectomy. Abdomen pelvic ultrasound and pelvic X-ray revealed a urinary bladder calculus. Examination of the previous radical prostatectomy scar found him to have a hypertrophic scar.

He had urethroscopy with bladder neck incision for bladder neck stenosis and cystolithotomy with resolution of the symptoms.

**Clinical discussion:**

The presentation was that of dysuria and frequency three years after radical prostatectomy. The cause of the symptoms was diagnosed after an abdomen pelvic ultrasound and pelvic X-ray as multiple bladder calculi. This is a rare finding with the few reported cases associated with clips that migrated to the urinary bladder forming a nidus for the calculi. This was of consideration in the case presented, however, the findings at urethroscopy revealed bladder neck stenosis suggesting stasis as possible cause of the bladder calculi. The symptoms resolved after bladder neck incision and cystolithotomy.

**Conclusion:**

In addition to clips forming a nidus for calculi in the urinary bladder after radical prostatectomy, bladder neck stenosis being the cause of urinary bladder calculi should be considered in a patient with hypertrophic scar.

## Highlights

• Bladder calculi after radical prostatectomy is rare and usually associated with migrated clips into the bladder.
• In patients with hypertrophic scars, there is the potential to form thick fibrosis at the reconstructed bladder neck
• The resulting urinary stasis may lead to calculi formation in the urinary bladder.
• Cystolithotomy, following bladder neck incision, preserves the integrity of the sphincter mechanism

## 1 Introduction

Post radical prostatectomy for localized prostate cancer with finding of bladder calculi is rare [1]. Reports indicate that such calculi arise from migration of clips into the urinary bladder forming a nidus for stone formation [1] [2].

The anastomotic site of the urethra with the urinary bladder after radical prostatectomy can undergo stenosis (bladder neck stenosis) during healing [3]. This leads to worsening lower urinary symptoms, and significant urinary stasis that can lead to bladder calculi formation from sedimentation or recurrent infection. Wound healing occurs in a dynamic process of balanced regulation. When the regulation is unbalanced leads to Hypertrophic scars and keloids. Both hypertrophic and keloidal scars are raised and firm as a result of over production of fibrinogen and collagen leading to raised, thickened scars. [4] [5] [6].

We present a case of multiple bladder calculi post radical prostatectomy for prostate cancer resulting from bladder neck stenosis in a patient with hypertrophic scar managed at an academic institution. This case is to bring to the attention of practitioners the need to consider bladder neck stenosis as a possible cause of bladder calculi post radical prostatectomy aside migrated clips in patients with abnormal scars.

## 2 Case report

A 60 year old man of African ancestry presented with retention of urine in April 2020. Further evaluation revealed a PSA of 88 ng/ml, a clinical T2b with histological confirmation adenocarcinoma of prostate Gleason score 7 (3 + 4). A whole-body MRI scan did not show any bone metastasis but reported pelvic lymphadenopathy. He opted for a radical prostatectomy with lymphadenectomy on account of the lower urinary tract symptoms which he had in May 2020. The histology confirmed adenocarcinoma of the prostate Gleason score 7 (3 + 4) and pathological T3b.

He was counseled for adjuvant radiotherapy with antiandrogen treatment but opted for anti-androgen treatment (goserelin injection 10.8 mg every three months), which he had for 12 months but stopped on account of insomnia while on the injections. The nadir tPSA was 0.03 ng/ml.

He presented with recurrence of lower urinary symptoms 3 years (April 2023) after the procedure with dysuria and urinary urgency at the outpatient clinic after defaulting planned regular follow up. There were no obstructive urinary symptoms, and the urinary bladder was not distended on examination. The tPSA was 0.32 ng/ml.

An abdomen pelvic ultrasound scan done revealed the presence of a bladder calculi with a post void residual urine of 8mls.

**Fig. 1:**
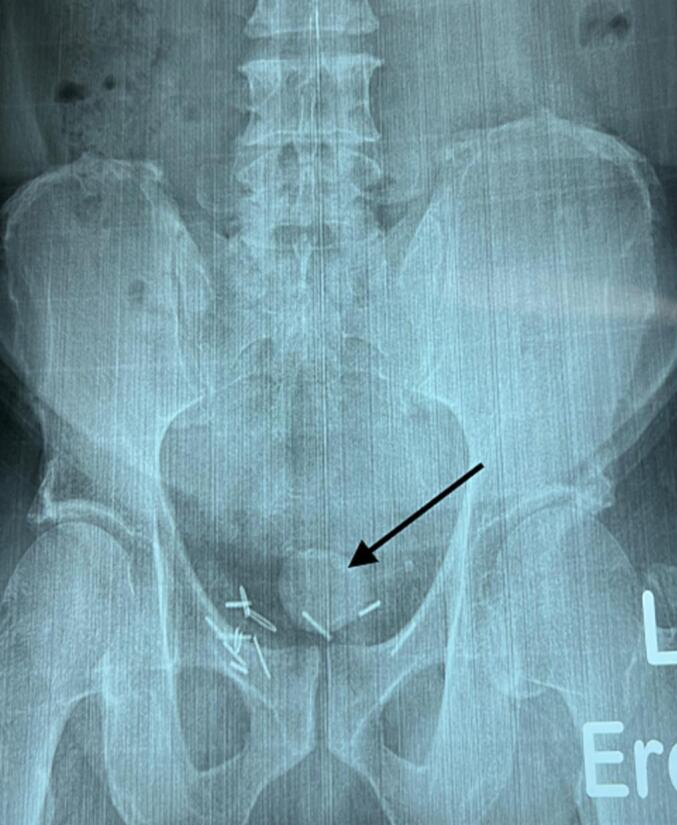
Pelvic X-ray showing bladder calculi (arrowed) and the clips used at the time of the radical prostatectomy.

A pelvic X-ray revealed a large bladder calculus (Fig. 1).

Patient was counseled for urethrocystoscopy and possible endoscopic fragmentation of the bladder calculi under spinal anesthesia.

**Fig. 2:**
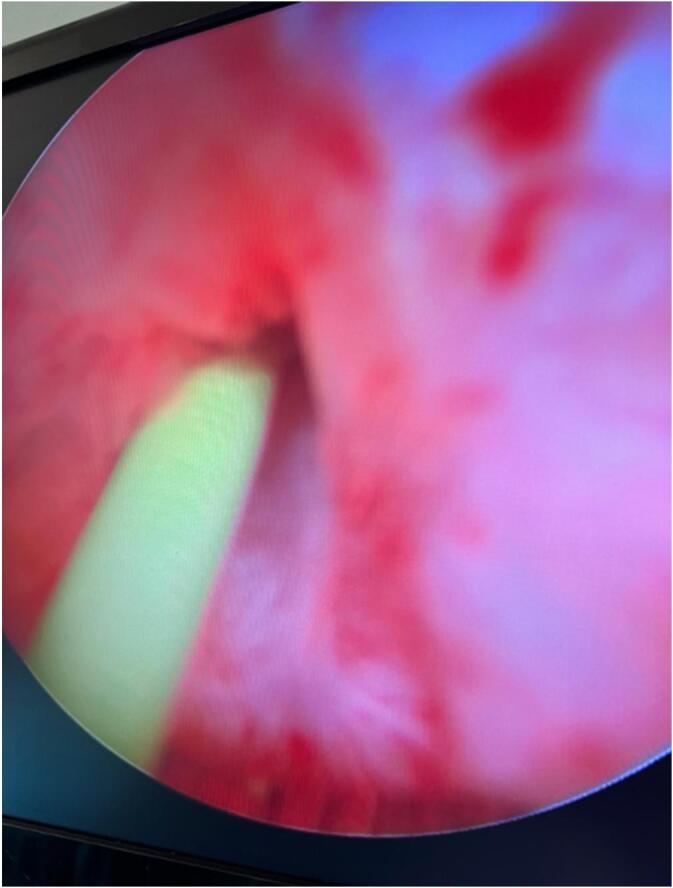
Dense urinary bladder neck stenosis admitting a guide wire.

At urethroscopy, a dense bladder neck stenosis was encountered that admitted the guide wire confirming possible stasis as the cause of the calculi formation in the urinary bladder (Fig. 2).

**Fig. 3:**
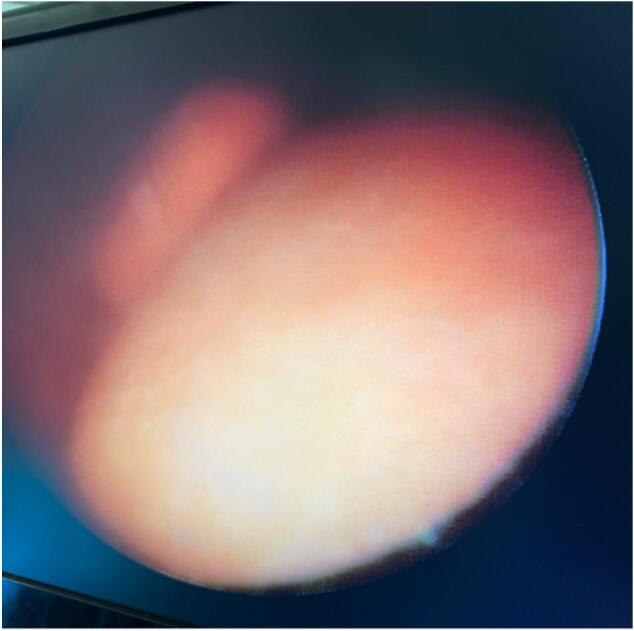
Cystoscopy with finding of calculi in the urinary bladder.

An incision of the bladder neck stenosis was done allowing for cystoscopy to be performed with the finding of three bladder calculi (Fig. 3).

**Fig. 4:**
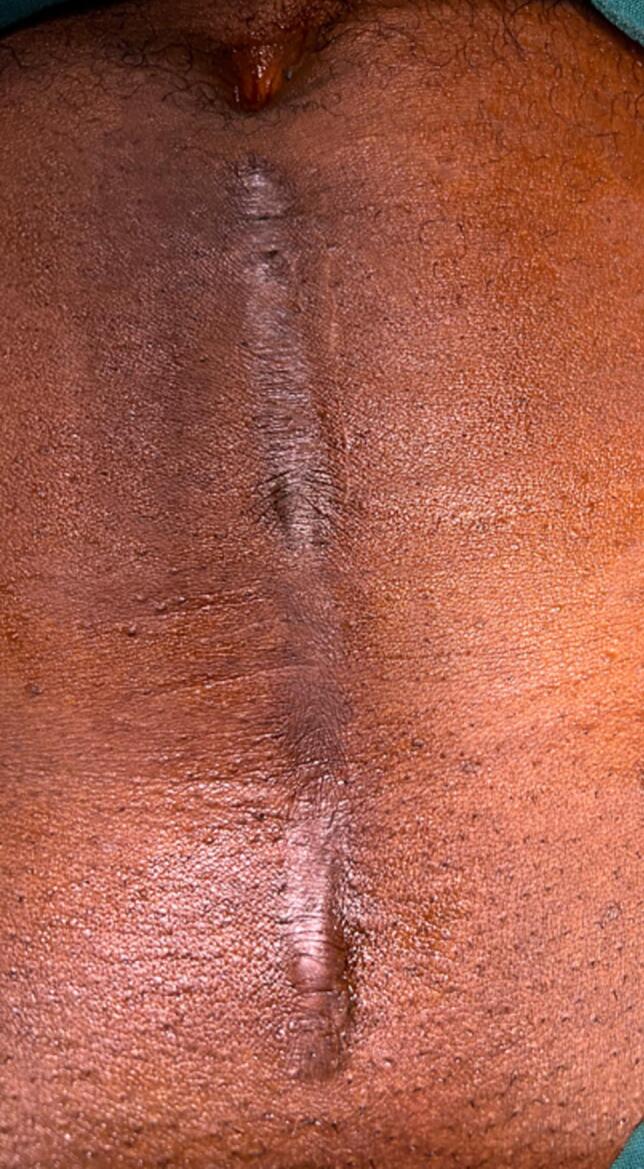
Hypertrophic scar from previous lower midline incision for radical prostatectomy.

A decision was taken to have an open cystolithotomy to avoid further disruption of the bladder neck and so an 18F foley catheter was passed for continuous bladder drainage. A careful inspection revealed hypertrophic scar of the previous incision used for the radical prostatectomy. This had not regress since appearing some months after the procedure. An over production of collagen was considered as a possible aetiology of the dense bladder neck stenosis (Fig. 4).

**Fig. 5:**
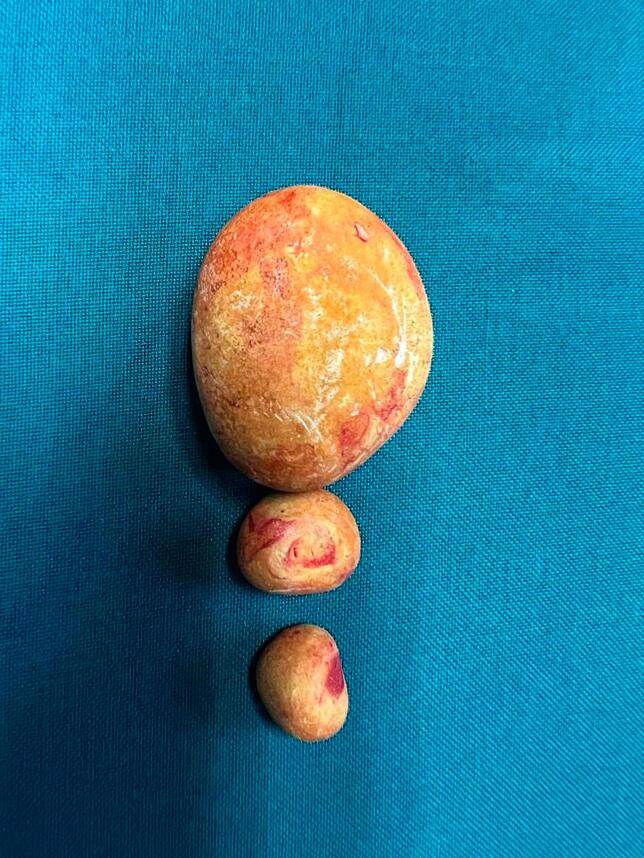
Three calculi retrieved from the urinary bladder: 4 cm, 2 cm and 2 cm as the sizes.

At cystolithotomy, the abnormal surgical scar was excised this was however not sent for histology. Three calculi were retrieved. The largest was 4 cm and two smaller- 2 cm calculi (Fig. 5). None of the calculi had clips as a nidus.

The urinary bladder was closed in two layers using vicryl 1 suture and the skin closed with Nylon 2–0.

The composition of a calculus was calcium oxalate monohydrate (20 %), Uric acid anhydrous (70 %) and Uric acid dihydrate (10 %) on analysis.

The patient recovered well and was discharged post of day 4 and the urethra catheter removed on the post operative day 14. He had been voiding well with no dysuria or frequency and was continent of urine and was satisfied with the outcome one month after removal of the urethral catheter. A follow up whole body MRI, the staging and imaging modality available, has been requested on account of the current PSA of 0.32 ng/ml to direct further management.

The work has been reported in line with the SCARE 2020 criteria [7].

## 3 Discussion

A complication of radical prostate is the formation of bladder neck stenosis. The incidence of bladder neck stenosis post open radical prostatectomy has been reported as 5.7 % [2]. The patients usually present with lower urinary tract symptoms, and they have been observed to respond to endoscopic treatments such as dilatation, direct visual internal urethrotomy or bladder neck incision. [8] The patient after the radical prostatectomy had no obstructive symptoms and was continent post procedure. The presentation was that of dysuria and frequency three years after the initial procedure. The cause of the symptoms was diagnosed after an abdomen pelvic ultrasound and KUB as bladder calculi post radical prostatectomy.

This is a rare finding with few reports. [1] [9] [10] [11]. The cases reported had been associated with clips that migrated to the urinary bladder forming a nidus for the calculi. This was of consideration in the case presented, however, the findings at urethroscopy revealed bladder neck stenosis suggesting stasis as possible cause of the bladder calculi. No loose clips were seen in the urinary bladder or associated with any of the three calculi retrieved. Thus, while migration of clips is to be considered in patients with bladder calculi post radical prostatectomy, a bladder neck stenosis is also to be considered. Bladder neck stenosis results in lower urinary tract symptoms or retention of urine [12] even in the absence of obstructive urinary symptoms.

The operative risk of developing bladder neck stenosis has been observed to include tension at site of anastomosis, post operative haemorrhage, large volume blood loss, pelvic haematoma formation and urinary leakage at site of anastomosis which may related to surgeons experience, prolonged catheterization, over narrowing of the bladder neck during anastomosis and acute urinary retention after urethral catheter removal [[12], [13], [14]] these are noted to cause peri-anastomotic inflammatory response leading to scar formation [[12], [13], [14]]. Pre-operative factors include obesity, diabetes mellitus, smoking, advanced age, hypertension, vascular disease and radiation therapy as these lead to poor microvascular environment that does not support or prolong anastomotic healing [12,13]. These factors lead to bladder neck stenosis within 6 months and hardly after 2 years [12,13]. The patient had no comorbidities.

The relatively dense fibrosis at the bladder neck with presence of hypertrophic scar supported the tendency of the patient to dense fibrosis which has been attributed to an overactivation of proliferation and migration with resultant disordered fibrosis and scaring. [4] [5] [6] This risk to the development of bladder neck stenosis post radical prostatectomy has been reported by Selvi et al. [13].

Patients with prior abnormal scars, need to have a wider bladder neck at reconstruction after radical prostatectomy as the dense fibrosis and contraction ultimately narrows the reconstructed bladder neck. There may be the need to keep the catheter in longer after the radical prostatectomy to help keep the bladder neck patent, however the effectiveness of such a measure may be questionable as the scar in this patient had not regress at three years. Hypertrophic scars compared to keloidal scars tend to be confined to the borders of the original wound appearing within 1 month of the wound and regressing after six months. Comparatively, dense fibrosis of keloidal scars progresses over months to years. [5,6]. Histologically, hypertrophic scars have collagen fibers being finer, arranged in parallel pattern and having a greater number of Type III collagen compared with type I collagen. Hypertrophic scars have myofibroblasts and alpha-smooth muscle actin. Keloid tissue on the other hand have abundance of collagen, arranged in a haphazard pattern or whorls, with an increased ratio of type I/III collagen [5]. The diagnosis of the hypertrophic scar in this case was by the physical findings (Fig. 4). Histology was not done. Since dense scaring can occur over months and even years in patients with abnormal scars especially keloids, careful follow up is needed after radical prostatectomy to identify this sequela for timely intervention. Other preventive measures to avoid bladder neck stenosis include the use of Robot assisted or laparoscopic procedures in vesico urethral anastomosis, the use of running sutures and improving the surgical technique in open procedures to avoid some of the risk factors. [14]

Like reports of successful endoscopic management of the bladder neck stenosis [8], the bladder neck incision was successful after passing a guide wire. In situations of recurrent bladder neck stenosis and dense fibrosis, adjuncts have been used to improve the outcome. These adjuncts include intralesional injection triamcinolone and mitomycin C [8]. There have been reports on the use of oral steroids such as deflazocort to prevent urethral stricture recurrence after optical internal urethrotomy. [15] No adjunct intralesional agent injection was performed in this patient.

Open cystolithotomy was adopted for the removal of the calculi against the use of an endoscopic lithotripsy procedure such as use of laser, ballistic or ultrasound [1] [10] to avoid further disruption of the bladder neck as bladder neck incision had been done. This was because it was anticipated the duration of an endoscopic procedure might be long due to the size and number of stones leading to possible injury to the bladder neck and the urethra. This choice appeared satisfactory as the patient maintained good urinary flow and continence after the procedure.

Though the patient had successful bladder neck incision and retrieval of the calculi, he will be followed up closely for possible recurrence of the bladder neck stenosis as the fibrosis was dense.

A follow up whole body MRI, the staging and imaging modality available, has been requested on account of the current PSA of 0.32 ng/ml to evaluate the prostatic bed as well as possible distant metastasis to direct further management of the prostate cancer.

## 4 Conclusion

Bladder calculus formation post radical prostatectomy is rare. Most reports associate its formation with migrated clips into the urinary bladder. Bladder neck stenosis with urinary stasis as an underlying cause should be considered when multiple and in a patient with a hypertrophic scar which has been noted to be one of the risk factors for developing bladder neck stenosis post radical prostatectomy. Such patients need to have long term follow up after lower urinary tract reconstruction procedures.

## Consent

Written informed consent was obtained from the patient for publication of this case report and accompanying images. A copy of the written consent is available for review by the Editor-in-Chief of this journal on request.

## Sources of funding

There was no source of funding. The authors are funding the work.

## Ethical approval

Case reports are exempt from ethical approval from our institution.

The patient has given written consent for the publication of this manuscript. No identifying features have been included in the manuscript.

An exemption was provided by the Ethical and Protocol Review Committee of the University of Ghana, College of Health Sciences, Korle Bu, Accra, Ghana.

## Registration of research studies

1. Name of the registry: Not Applicable.
2. Unique identifying number or registration ID:
3. Hyperlink to your specific registration (must be publicly accessible and will be checked):

## CRediT authorship contribution statement

**Mathew Yamoah Kyei**; Study concept or design, patient treatment planning, data collection, data interpretation, writing the paper, review and consent for final publication of the manuscript.

**Maxwell Agyapong Nyinah**; Study concept or design, patient treatment planning, data collection, data interpretation, writing the paper, review, and consent for final publication of the manuscript.

**Ali Ayamba**; Study concept or design, patient treatment planning, data collection, data interpretation, review, and consent for final publication of the manuscript.

**Josephine Mpomaa Kyei**; Study concept or design, patient treatment planning, data collection, review, and consent for final publication of the manuscript.

**James Edward Mensah**; Study concept or design, patient treatment planning, data interpretation, review, and consent for final publication of the manuscript.

## Guarantor

Mathew Yamoah Kyei.

## Declaration of competing interest

The authors have no conflict of interest to declare.
